# Biosensors for Studies on Adhesion-Mediated Cellular Responses to Their Microenvironment

**DOI:** 10.3389/fbioe.2020.597950

**Published:** 2020-11-11

**Authors:** Nicolás Andrés Saffioti, Elisabetta Ada Cavalcanti-Adam, Diego Pallarola

**Affiliations:** ^1^Instituto de Nanosistemas, Universidad Nacional de General San Martín, San Martín, Argentina; ^2^Department of Cellular Biophysics, Max Planck Institute for Medical Research, Heidelberg, Germany

**Keywords:** cell adhesion, electrochemistry, optical microscopy, mechanobiology, integrins, biomaterials, cell–cell adhesion, extracellular matrix

## Abstract

Cells interact with their microenvironment by constantly sensing mechanical and chemical cues converting them into biochemical signals. These processes allow cells to respond and adapt to changes in their environment, and are crucial for most cellular functions. Understanding the mechanism underlying this complex interplay at the cell-matrix interface is of fundamental value to decipher key biochemical and mechanical factors regulating cell fate. The combination of material science and surface chemistry aided in the creation of controllable environments to study cell mechanosensing and mechanotransduction. Biologically inspired materials tailored with specific bioactive molecules, desired physical properties and tunable topography have emerged as suitable tools to study cell behavior. Among these materials, synthetic cell interfaces with built-in sensing capabilities are highly advantageous to measure biophysical and biochemical interaction between cells and their environment. In this review, we discuss the design of micro and nanostructured biomaterials engineered not only to mimic the structure, properties, and function of the cellular microenvironment, but also to obtain quantitative information on how cells sense and probe specific adhesive cues from the extracellular domain. This type of responsive biointerfaces provides a readout of mechanics, biochemistry, and electrical activity in real time allowing observation of cellular processes with molecular specificity. Specifically designed sensors based on advanced optical and electrochemical readout are discussed. We further provide an insight into the emerging role of multifunctional micro and nanosensors to control and monitor cell functions by means of material design.

## Introduction

Cell adhesion is a critical aspect of the constitution of tissues and organs. The complex organization of tissues relies on a precise control over the formation of adhesive contacts between cells and the extracellular matrix (ECM) (Gumbiner, 1996). From regenerative medicine to development biology there is a great interest in comprehending the mechanisms that control the assembly of cells into tissues and organs (Keung et al., 2010; Gaharwar et al., 2020). Increasing evidence has shown that these processes are regulated not only by biochemical signals but also by biophysical cues from the environment. For example, it has been found that cells are able to sense and respond to the topography (Curtis and Riehle, 2001; Spatz and Geiger, 2007), rigidity (Discher et al., 2005; Engler et al., 2006), and anisotropy (Théry et al., 2006; Xia et al., 2008) of their environment.

To reveal the mechanosensory elements involved in cell-cell and cell-ECM interactions, ECM-inspired materials have been developed (Mager et al., 2011; Xi et al., 2019). Advancements in material science and surface chemistry made it possible to create materials that mimic both physical (e.g., stiffness and topography) and chemical cues (adhesive and soluble) of the extracellular environment. These materials are highly desired because cell development and behavior can be studied under conditions similar to those found in the cell microenvironment *in vivo*. As materials incorporate biochemical and biophysical cues from the natural ECM, the information they provide allows a closer estimation of the *in vivo* situation. Among biomaterials, those with built-in sensing properties are particularly attractive to obtain quantitative information on how cells probe and respond to relevant physicochemical cues of the ECM. The pioneering work of Dembo and Wang (1999) on deformable elastic materials with embedded fluorescent tracers was one the first material of this class capable of deciphering cell contractile forces. Similarly, in the field of electrochemical sensors, the work of Giaever and Keese (1984, 1986, 1991) set the foundations for the development of materials capable of probing the cell adhesion interface in real time. Both approaches profited from their label-free capabilities. Since then, a myriad of synthetic responsive biointerfaces with unprecedented temporal and spatial resolution was engineered to study cell signaling and behavior in real time and with high sensitivity.

In this review, we will focus on the design of micro and nanostructured biomaterials that resemble the complex properties of the ECM, and that play an active role in measuring cell-adhesion related processes. Materials developed to study the impact of different properties of the ECM on cell behavior, but only provide adhesion support for cells are covered elsewhere (Mager et al., 2011; Rosales and Anseth, 2016). Emphasis will be placed on label-free sensing schemes. These approaches have advantages over those that require labeling. Label-free sensors offer real-time measurements, less or no sample preparation, and low non-specific response, reducing the risk of generating artifacts and false positives in the measurements. Commonly used labels like fluorescent or colorimetric dyes are often cytotoxic and hamper further use of the cultured cells, which is particularly desired for regenerative tissue applications. Thus, biomaterials with sensing capabilities hold great potential to bridge the gap between traditional cell binding assays and *in vivo* studies.

We aim to offer readers an overview of the latest sensing biomaterials and their main advantages and applications in order to guide the selection of the most appropriate platforms for specific purposes. We will describe the specific features that have been provided to materials and how these characteristics have contributed to reveal key aspects of the cellular adhesion mechanism. Although most of the systems described in this review are research-oriented, commercial applications are possible especially in biomedical diagnosis (Suhito et al., 2018; Tutar et al., 2019) and tissue engineering (Mitrousis et al., 2018; Gaharwar et al., 2020).

## Cells Adhesion to the Microenvironment

**FIGURE 1:**
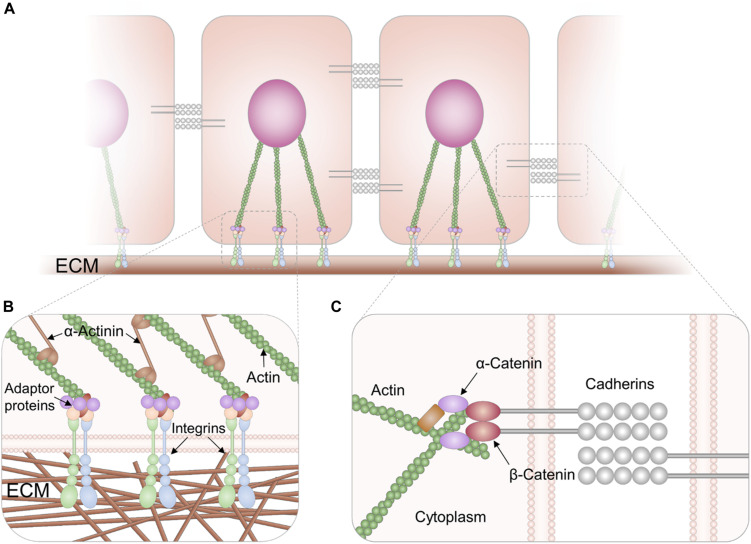
Molecular complexes involved in cell adhesion processes. Cells form bonds with the ECM and with other cells by protein receptors in the plasma membrane **(A)**. These receptors are linked to the cytoskeleton by adaptor proteins. In **(B)**, a scheme of a FA where integrins (formed by α and β subunits) recognize specific ligands in the ECM. In the cytoplasm, talin, kindling, and vinculin among other proteins (“adaptor proteins” in the image) associate with the integrins forming a complex that acts as a molecular clutch. The adaptor proteins connect the integrins with the cytoskeleton and participate actively in mechanosensing. In **(C)**, a scheme of an adherent junction where cadherins in the extracellular medium establish bonds with cadherins in the other cell. The link between cadherins and the actin cytoskeleton is mediated by adaptor proteins α and β catenins.

The ECM is a complex and dynamic mixture of proteins and polysaccharides that provides not only support for cells but also is involved in regulating many important cellular processes including proliferation, survival, differentiation, and apoptosis (Frantz et al., 2010). Cells sense and respond to changes in topological, physical, and chemical properties of the ECM through a sophisticated system that allows them to adapt their behavior by converting these cues into biochemical signals. The interaction of cells with the ECM is mostly mediated by a family of cell transmembrane receptors called integrins that are responsible for cell attachment, and connect the cell-matrix adhesions with the cell cytoskeleton (Geiger and Yamada, 2011) (Figure 1A). Integrins undergo conformational changes upon biochemical and mechanical interactions leading to outside-in and inside-out mechanotransduction. Although it is well known that integrins have a crucial role in regulating diverse adhesion-related functions, the mechanism by which cells translate extracellular stimuli into biological responses remains unclear.

Integrins are heterodimers constituted by two transmembrane protein subunits α and β which bind to specific ligands located in the ECM proteins or in membrane of other cells (I-CAM and V-CAM receptors) (Sun et al., 2019). Integrins adopt closed or open conformations characterized by low or high-affinity states, respectively. The transition from the closed to the open conformation is crucial for integrin activation and can be induced from the extracellular medium or from the cytoplasm (Luo et al., 2007). Upon activation, integrins form clusters and associate with adaptor proteins like talin, kindling, and vinculin among others. These proteins connect the integrins to *F*-actin fibers forming a molecular “clutch” that mediates mechanical forces between the membrane and the cytoskeleton (Figure 1B) (Sun et al., 2019). The macromolecular complex of integrins and adaptor proteins constitute the focal adhesions (FA). It has been shown that FA act as mechanosensory machines, translating multiple environmental cues to cellular responses (Geiger et al., 2009). The integrin-mediated binding to the ECM, even though being one of the most important adhesion mechanisms is not the only one. Syndecans and lectins also participate in cell adhesion, although their role in mechanosensing is not totally clear (Gumbiner, 1996; Mager et al., 2011; Guilluy and Dolega, 2020).

Cell–cell contacts are mediated by different types of junctions: adherent junctions, tight junctions, and desmosomes (Gumbiner, 1996). Adherent junctions are one of the most important sites of intercellular mechanical coupling (Ladoux and Mège, 2017). Cadherins are integral membrane proteins that participate in the formation of adherent junctions. Their extracellular domains mediate the adhesion to neighbor cells, whereas their intracellular regions are connected to the actin cytoskeleton by adaptor proteins α and β-catenin (Figure 1C). Cadherins associate between them and with adaptor proteins to form clusters constituting the mature adherent junction (Mège and Ishiyama, 2017). Cadherin complexes respond to tension load by the actomyosin. When pulling forces are applied to the adherent junction through the actin cytoskeleton, α catenin unfolds and associates with vinculin, which strengthens the adherent junction (Le Duc et al., 2010; Yonemura et al., 2010; Buckley et al., 2014). Therefore, mechanosensing in cell-cell bonds is mediated by the same structures that mediate cell–cell adhesions. There is also evidence that tight junctions mediate mechanosensing in epithelia by association with the actin cytoskeleton (Tornavaca et al., 2015). Desmosomes are tightly associated with intermediary filaments and participate in mechanosensing (Weber et al., 2012), although their role in mechanotransduction is complex as intermediary filament proteins are diverse and their expression is tissue specific (Ladoux and Mège, 2017). An excellent review on cell-to-cell association and the dynamics of collective cell behaviors has been published by Ladoux and Mège (2017).

Cell-to-cell adhesion is also important in cell communication processes. During antigen presentation, antigen presenting cells (APC) associate with T or B lymphocytes forming the immunological synapse. Evidence has shown that this process is mechanosensitive, although cell-to-cell bonds and mechanosensing is mediated by the interaction of the peptide major histocompatibility complex (pMHC) in the APC with the specific T or B cell receptor in the lymphocyte (Bashoura et al., 2014; Liu B. et al., 2014; Liu et al., 2016).

## Relevance of Biosensors in Cell Adhesion Studies

Much of the information we have so far on how cells adhere to the ECM and other cells have come from a wide set of bioanalytical tools. Genetic modification like gene knockout (Elosegui-Artola et al., 2016; Strohmeyer et al., 2017), protein expression modulation by siRNA (Plotnikov et al., 2012; Bazellières et al., 2015) and the use of externally added inhibitors (Bashoura et al., 2014; Collins et al., 2017) are important methods to investigate the role of different proteins in the cell sensing mechanism. Cell behavior as a consequence of these modifications is often monitored by optical microscopy-based techniques like immunofluorescence (Engler et al., 2006), *in vivo* observation of recombinant fluorescence proteins (Reffay et al., 2014; Oria et al., 2017), genetically incorporated molecular probes (Grashoff et al., 2010), and more recently optochemical probes (Endo et al., 2019; Ollech et al., 2020). Even though these methods have revealed important aspects of the molecular mechanisms of cellular mechanotransduction, they could not provide a complete description of the interaction between cells and their environment. In this sense, biomaterials with built-in sensing capabilities have contributed to our understanding of cell mechanosensing by providing information not previously available by other bioanalytical methods. For example, it was not until the work of Harris et al. (1980) with soft silicon substrates that traction forces generated by cells were quantified during cell spreading and migration. Decades later, advances in material science and polymer chemistry have enabled remarkable improvements of this groundbreaking approach leading to a better understanding of how cells mechanically interact with the ECM (Roca-Cusachs et al., 2017).

Noteworthy, although optical microscopy-based techniques have been fundamental for our understanding of the cell adhesion process, they often involve endpoint measurements and time-consuming sample preparation (e.g., immunostaining). This greatly restricts the temporal resolution achievable with these techniques. Genetically encoded fluorophores can overcome this drawback but require modification of cell genome, and the time span of the measurement is limited by photobleaching. Transillumination microscopy imaging like phase contrast or differential interference contrast does not have this limitation, but provide only qualitative information. In this context, non-invasive and non-destructive methods stand out as sensitive and quantitative approaches to study cell-adhesion related processes in real-time. These include electrochemistry, quartz crystal microbalance (QCM), surface enhanced Raman spectroscopy (SERS), and surface plasmon resonance (SPR) (Janshoff et al., 2010; Méjard et al., 2014; Suhito et al., 2018) (More details of these approaches can be found in sections “Cell-substrate adhesions” and “Cell adhesion biomarkers”). Analytical approaches based on these techniques provide unique information with high time resolution about the cell-matrix and cell-cell interface. For instance, electric cell-impedance sensing (ECIS) methods can easily reveal the formation of cell–cell junctions and cell-substrate contacts, and are extensively used to follow epithelial maturation (Ngok et al., 2013; Gamal et al., 2017; Van Der Stoel et al., 2020). Surface enhanced Raman spectroscopy (SERS) is ideal to obtain information about biochemical changes inside cells in the proximity of a nanostructured surface (El-Said et al., 2015; Haldavnekar et al., 2018; Rusciano et al., 2019).

In cell culture, substrate materials provide the physical scaffold that supports cells allowing their adhesion and proliferation. Therefore, these materials have to be biocompatible, meaning that they have to comply with determined characteristics in terms of topology, stiffness, and chemical composition. However, substrates are not necessarily relegated to being a physical support, on the contrary, by incorporating a transducer element these materials can reveal crucial aspects that regulate cell behavior. This type of responsive biomaterials can be classified as biosensors. Conventionally, biosensors are devices able to provide selective quantitative analytical information using a biological recognition element and a transducer component. In the context of this review, the recognition element is often a specific cell-binding molecule (e.g., the tripeptide motif Arg-Gly-Asp (RGD) to bind α_v_β_3_ integrin), while the transducer can be optical (SERS, traction force microscopy), electrochemical (ECIS), or piezoelectric (QCM). Because of its pivoting role, the transducer element will define important aspects of the analytical response as spatial and time resolution, signal to noise ratio, and selectivity.

Despite all the progress made in the study on how cells sense and react to physicochemical properties of their environment, understanding the mechanisms by which cells can transduce mechanical signals into biochemical events is still a challenge. Moreover, the relationship between these events and cell differentiation, physiological function, and pathology have not been elucidated (Mohammed et al., 2019). In the field of regenerative medicine, a scenario like this raises important questions on how to ensure the efficiency of materials to promote cell differentiation. In this regard, biomaterials with cell-instructive characteristics and built-in sensing capabilities could provide valuable information about cell-material interaction. Devices capable of providing new sources of information will be key to elucidate how different properties of the cell matrix affect cell behavior. In order to discuss the relevance of the biosensors in cell behavior studies, each section of the review nucleates biosensors in terms of the biological variable about which they provide information.

## Biological Applications of Biosensors

### Cell-Substrate Adhesions

After a cell in suspension makes the first contact with a substrate, it carries out successive steps of attachment, adhesion, spreading, and in some cases followed by migration and proliferation. To allow cell adhesion, substrates require the presence of adhesion-promoting proteins or ligands immobilized on the substrate (Janshoff et al., 2010). There are two options to achieve substrate modification to elicit cell adhesion: the adhesive molecules are incorporated during the substrate synthesis or they are secreted by the cells in the adhesion process. In the second case, the material has to allow protein absorption. Surface wettability and topography have a major influence on this process (Prime and Whitesides, 1991; Janshoff et al., 2010).

Most cells have excellent insulating properties. When cells adhere on an electrode surface, they modify the environment at the solution-electrode interface affecting the charge transfer events at the surface (Giaever and Keese, 1993; Ding et al., 2008). This phenomenon was exploited by Giaever and Keese (1984, 1991) when they created the first electrodes to study cell adhesion using electric impedance. In their design, the substrate incorporated working electrodes and a counter electrode. The working and counter electrodes were connected to a lock-in amplifier, and the culture medium completed the circuit. The authors monitored cell adhesion events by applying an alternating sinusoidal voltage and monitoring current. When cells adhered and spread on the working electrode they generated an impedance increase as a consequence of the formation of an insulating layer (Giaever and Keese, 1993). This technique was called electric cell impedance sensing (ECIS) and due to its high sensitivity, it has been used for monitoring cells attachment (Han et al., 2011; Xue et al., 2011), spreading (Wegener et al., 2000; Arias et al., 2010; Pradhan et al., 2014), locomotion (Giaever and Keese, 1991; Wang et al., 2008), and apoptosis (Arndt et al., 2004; Liu et al., 2009). Impedance measurements depend on the number of cells seeded on the electrode, their morphology, motility, and on the formation of cell–cell interactions (for further details see section “Cell-Cell adhesion”). Analysis of data is aided by mathematical models that allow calculating cell morphological parameters (Giaever and Keese, 1991; Lo et al., 1995). For more detailed reviews on ECIS please see Janshoff et al. (2010) and Hong et al. (2011). The use of ECIS substrates for monitoring cell behavior has advantages compared to traditional optical microscopy methods. ECIS is a non-invasive and non-destructive technique capable of providing information without the needing for cell staining (Suhito et al., 2018). In microscopy, quantification of the cell adhesion and spreading requires tedious data processing compared to the straightforward information provided by impedance measurements. Besides, impedance can be registered on cells over days with a temporal resolution of seconds (Hong et al., 2011). Moreover, transparent ECIS substrates can be excellent complements to optical microscopy as ECIS can provide information not easily accessible to visualization like the formation of cell-cell junctions, or cell micromotion (Giaever and Keese, 1991; Lo et al., 1995) (Table 1).

**TABLE 1:** Comparison between label-free methods to measure cell-substrate adhesion.

**Method**	**Principle for monitoring cell adhesion**	**Advantages**	**Disadvantages**	**Resolution**	**References**
ECIS	Changes in the impedance at the electrode-medium interface	• Cell-surface or cell-cell adhesion monitoring depending on current frequency • Sensing surface can be controlled with electrode design • Surface can be modified with micro and nanostructures	• Obtaining cell morphological parameters requires mathematical modeling	• Classical ECIS senses bulk cell population (no spatial resolution) • Single cell resolution using MEA • Spatial sub μmeter resolution in combination with SPR or optical microscopy.	Giaever and Keese, 1993; Lo et al., 1995; Ding et al., 2008; Wang et al., 2011; Susloparova et al., 2015
QCM	Changes in resonance frequency of the piezoelectric substrate	• Changes in ΔD/Δf ratio as a fingerprint for cell-surface adhesion process • Versatile chemical functionalization of the substrate	• Non-rigid coatings complicates interpretation of data • Lack of direct correlation between signal and cell morphology	• Bulk cell population (no spatial resolution)	Fredriksson et al., 1998; Janshoff et al., 2010; Xi and Chen, 2013; Zhang et al., 2018
SPR	Changes in the refractive index of the substrate surface	• High sensitivity to events within 200 nm of the surface • Sensitive to cell morphology	• Low sensitivity to events 200 nm above the surface • Limited to thin coatings • Relative more expensive	• Classical SPR senses bulk cell population (no spatial resolution) • SPR microscopy allows μmeter resolution	Rothenhäusler and Knoll, 1988; Willets and Van Duyne, 2007; Chabot et al., 2009

**FIGURE 2:**
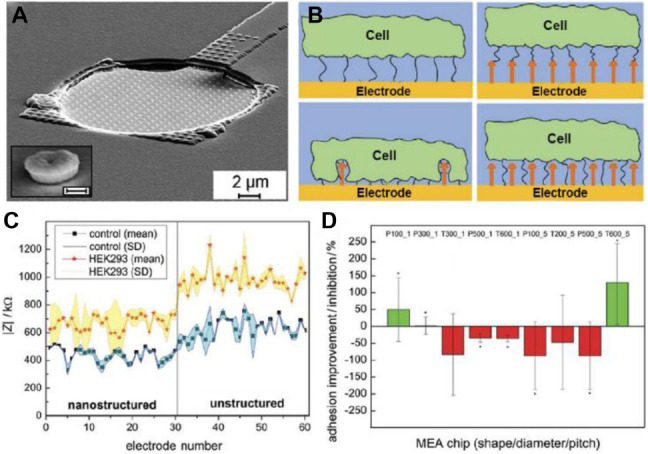
A nanostructured biosensor for the detection of cell-adhesion. In **(A)**, a SEM image of a nanostructured electrode with incorporated nanostructures separated by 1 μm (inset: magnification of a tube-like nanostructure, scale bar: 200 nm). In **(B)**, the image shows, the possible cell adhesion mechanisms of the cell to the surfaces. Compared to an unstructured electrode (image at top left) the cell can bind to the top of the nanostructures increasing cell–electrode distance and lowering impedance (top right). Alternatively, they can promote cell adhesion resulting in a closer cell–electrode distance and higher impedance signals after cell adhesion (bottom left). Finally, it is possible that nanostructures have no effect on cell adhesion (bottom right). In **(C)**, a representative example of recordings showing the magnitude of impedance (| Z|), before (control) and after cell attachment on the nanostructured (no. 1–30), or unstructured electrodes (no. 31–60). In **(D)**, the normalized ratio of the increase/decrease of the impedance ΔZ for nanostructured electrodes compared to the unstructured ones. *P*-value < 0.05 compared to unstructured electrodes. Figures were adapted from Decker et al. (2019) with permission. Copyright 2019 John Wiley and Sons.

Coatings on substrate materials for ECIS can offer better control of adhesive cell behavior without hampering the sensing capabilities of the electrodes (Giaever and Keese, 1986). Different strategies, including adherent polymer coatings (Choi et al., 2015), self-assembled monolayers (SAM) (Parviz et al., 2017), metallic nanoparticles (Kim et al., 2013; Pallarola et al., 2017a), carbon nanotubes (Srinivasaraghavan et al., 2014), and silicon nanowires (Abiri et al., 2015) have been reported. Susloparova et al. (2015) created new substrates for ECIS using open gate field-effect transistors instead of gold electrodes, which allowed to obtain single-cell resolution of the impedance measurements. Decker et al. (2019) employed 3D nanostructured multielectrode arrays to study cell adhesion. Using nanoimprint lithography, the authors created an electrode with incorporated nanostructures in different forms, dimensions, or pitch lengths in a reproducible way. By changing the synthesis parameters, especially the time during electroplating, the height and shape of the nanostructures could be modulated (Figures 2A,B). The authors created a multi-electrode array with half of the electrodes with nanostructured patterns and half without them. The authors tested different type of nanostructures with a pillar shape modifying the distances between nanostructures and their shape. Cells could attach to both the nanopatterned and unpatterned electrodes, although the nanostructured ones displayed a lower impedance (Figure 2C). However, upon cell adhesion some nanostructures showed increased cell-nanostructure coupling and increased impedance change as consequence of cell adhesion (Figure 2D). This work showed how nanostructured topologies on electrodes could improve ECIS biosensing capabilities. Moreover, ECIS sensitivity could be enhanced using redox probes. In this approach, the probe (for example [Fe(CN)_6_]^3–^) is incorporated in the culture medium (Ding et al., 2008). Cell adhesion and spreading on the electrode forms a barrier that hinders the access of the probe to the electrode, decreasing electron transfer. Thus, high sensitivity to the area covered by the cell can be achieved (Ding et al., 2007). A modification of this strategy was used in a recent work from Du et al. (2020) where they created a biosensor using nanocomposite materials to follow the epithelial-mesenchymal transition of A459 lung cancer cells.

Piezoelectric materials can also be employed for monitoring cell adhesion. Quartz microbalance experiments are performed on sensor materials made of an α-quartz disk sandwiched between two metal electrodes. Due to the piezoelectric nature of α-quartz, any mechanical deformation of the crystal creates an electrical potential at the quartz surface, and vice versa (Janshoff et al., 2010; Chen et al., 2018). In most common approaches, an alternating current is applied between the electrodes allowing measuring the resonance frequency of the crystal over time. Cells can adhere and grow on the resonator surface, which produces changes in its resonance frequency (Δf). Moreover, other materials can be deposited on the resonator surface to assess the cell adhesion to them. It has been shown that Δf changes correlate with cell coverage on the sensor surface (Redepenning et al., 1993; Wegener et al., 1998; Tagaya et al., 2011). Hence, monitoring Δf as a function of time was employed to follow cell adhesion, spreading, and proliferation (Ishay et al., 2015). However, due to the viscous nature of cells and culture mediums, the changes in vibrational energy dissipation (ΔD) of the sensor can also provide relevant information on cell behavior. Nonetheless, the link between ΔD and the physical characteristics of cells that elicit these changes has not been understood (Xi and Chen, 2013). The ratio ΔD/Δf has been regarded as a fingerprint of the cell adhesion process, as different cell lines display different ΔD and Δf behaviors during adhesion and proliferation (Fredriksson et al., 1998). QCM sensors can be modified with coatings to provide enhanced cell adhesion, although the nature of the coating could influence the responses of the sensor (Lord et al., 2006). Despite the needing for non-common materials (α-quartz sensors), QCM is an inexpensive and valuable technique to monitor cell adhesion dynamics. Table 1 summarizes the advantages and disadvantages of QCM in cell adhesion study.

Materials with surface plasmonic properties allow the implementation of an evanescent wave-based optical technique, surface plasmon resonance (SPR). SPR can be employed for monitoring cell adhesion processes in the proximity of the substrate surface (Chabot et al., 2009; Peterson et al., 2009; Wang et al., 2012). SPR sensors consist of a glass substrate (LaSFN9 or BK7) coated with a thin gold layer (∼50-nm). These sensors allow monitoring cell-substrate interactions occurring in the proximity of the first few hundreds nanometers over the gold layer due to the evanescent decay of the plasmon perpendicular to the surface interface (Willets and Van Duyne, 2007). The molecules in the proximity of the interface interact with the confined electromagnetic wave, resulting in changes in the refractive index at the metal surface (Homola, 2003; Willets and Van Duyne, 2007). Therefore, events occurring on the substrate surface such as cell adhesion and spreading modify the local refractive index of the surface, which can be followed in real time (Yashunsky et al., 2010; Borile et al., 2019). The changes in the refractive index are measured by irradiating the gold surface through a high refractive index prism in an angle that yields total internal reflection. The reflected light is interrogated by varying the angle of incidence at a fixed wavelength or by changing the wavelength at a fixed angle (Willets and Van Duyne, 2007). Also, spatial resolution of the cell-substrate contacts can be obtained using SPR microscopy (Rothenhäusler and Knoll, 1988). SPR has the advantage of detecting changes in cell morphology, outperforming ECIS and QCM in this aspect (Table 1). Noteworthy, SPR can be combined with electrochemical measurements as the local effective refractive index on a determined spot depends on the local charge density of the surface. Changes in the electrode interface by cell adhesion processes modify the local impedance of the surface, which is translated into changes in the local SPR signal. Using this phenomenon, Wang et al. developed electrochemical impedance microscopy (EIM) using a transparent conductive substrate with plasmonic properties (Wang et al., 2011). Mapping the local impedance on the surface allowed obtaining high-resolution images of the cell-substrate contacts. This technology is an example of how recent advances in the use of biosensor substrates allow characterizing the cell-substrate interaction with increasing time and spatial resolution.

### Cell–Cell Adhesion

Direct interactions between cells are often mediated by a set of ligands and receptors expressed by both cells. When cells have to build long-term bonds between them, they assemble different types of junctions: adherent junctions, tight junctions, and desmosomes (Ladoux and Mège, 2017). In other cases, cell–cell adhesions have to be transient like those formed by the natural killers lymphocytes and their target cells (Orange, 2008).

**FIGURE 3:**
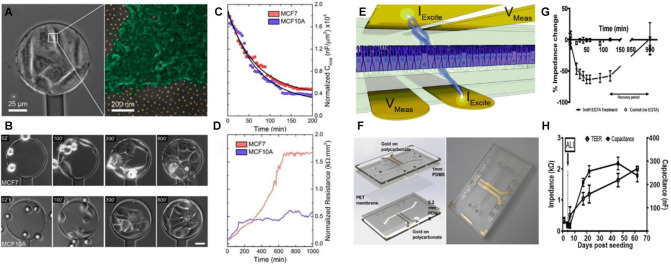
Biosensors based on electrochemical readout for monitoring cell-cell junctions. In **(A)**, the image on the left shows an optical microscopy image of a cell-coated sensor. The magnification shows a cell on a nanopatterned surface, where the golden dots correspond to the AuNP in a quasi-hexagonal array. In **(B)**, phase-contrast microscopy images show cell adhesion dynamics on a nanopatterned electrode (53 nm spacing between AuNP). AuNP were coated with specific α_v_β_3_-ligands. Scale bar: 30 μm. In **(C,D)**, the graphs show resistance and capacitance respectively, as a function of time for MCF7 and MCF10A cell lines. The results indicate that MCF7 cells induce a higher change in the electrode resistance in comparison with MCF10A cells. This can be attributed to the differential expression of E-cadherin. Figures **(A–D)** were adapted from Pallarola et al. (2017a) with permission. Copyright 2017 American Chemical Society. In **(E)**, the image shows a schematic representation of the device for measuring transepithelial electrical resistance. The *I*_Excite_ electrodes apply a small current of 10 μA and the V_Meas_ electrodes measure the respective potential across the cell epithelium. In **(F)**, the left panel shows a CAD model of the chip employed, while on the right panel a photograph of the assembled chip is shown. Gold electrodes were patterned on polycarbonate substrates. The polyethylene membrane where the cells adhered was placed between two PDMS layers that formed the microfluidic channels. The assembly was placed between two polycarbonate layers to assemble the chip. The graph in **(G)** shows the relative impedance change as a function of time of intestinal epithelial cells in the absence or presence of 5 mM EGTA. The decrease in impedance in the presence of EGTA was attributed to the disassembly of the cell to cell junctions. The graph in **(H)** shows the TEER and capacitance of a culture of airway epithelial cells as a function of time. In this case, the cell line was cultivated submerged in liquid medium on both sides of the epithelium. Then, on day 6, the liquid of the upper chamber was removed. Thus, an air-liquid interface was generated (ALI) emulating the natural environment of the cells. Figures **(E–H)** were adapted from Henry et al. (2017) with permission. Copyright 2017 Royal Society of Chemistry.

Recently, Pallarola et al. (2017a) developed a nanostructured electrochemical sensor exhibiting high sensitivity to the constitution of cell–cell adhesion interactions. The sensing platform consisted of a 100-μm-diameter ITO microelectrode patterned with an ordered array of AuNPs, and surrounded by a SiO_2_-insulating layer (Figure 3A). The sensor was built by a combination of diblock copolymer micelle nanolithography (Pallarola et al., 2014) and photolithography. The use of a gold nanopatterned surface allowed a precise control over the distribution of cell adhesion ligands on a non-adhesive PEG-passivated background (Pallarola et al., 2017b). Cell behavior was monitored over several hours by simultaneous electrochemical impedance spectroscopy and optical microscopy (Figure 3B). The surface of the electrode exhibited high sensitivity toward early events of cell interaction. Resistance and capacitance recordings were used to study the behavior of different cell types. It was observed that cell lines expressing lower levels of *E*-cadherins registered lower resistance values at low frequency (429 Hz) (Figures 3C,D). This was in agreement with the fact that increased cell-to-cell adhesion results in a lower paracellular current (Hong et al., 2011). This feature allowed for distinguishing between different cell types based on the density of adherent junctions, as observed for MCF-7 cells, in comparison with MCF-10A cells. This approach is a powerful tool to study the dynamics of cell–cell contact formation and remodeling of junctions under specifically engineered environments in a highly sensitive, instantaneous, and non-destructive manner.

The integrity of epithelia relies on the ability to form strong junctions between cells. Another suitable approach to monitor the formation of cell-cell contacts is to measure the electrical impedance across an epithelium placed between two electrodes. Measurements of *trans*-epithelial electrical resistance (TEER) is a valuable method for evaluating *in vitro* barrier tissue integrity. TEER measurements of cell cultures were widely utilized in research (Ferruzza et al., 1999; Huh et al., 2010; Lippmann et al., 2012) and for a more detailed review on this topic see the article of Srinivasan et al. (2015). In particular, the integration of immobilized TEER electrodes with microfluidics holds great potential to study cell barrier functions and cell behaviors in cell-mimetic environments. For example, Henry et al. (2017) developed a robust approach to fabricate organ-on-chip based on microfluidics with fully integrated electrodes (Figures 3E,F). The assembly of tight junctions was monitored by measuring the TEER and capacitance at low frequency. The authors demonstrated that when cells established tight junctions between them, capacitance reaches its maximum. A classical experiment to prove the ability of electrochemical devices to measure the formation of tight junctions is to add EGTA to the culture medium (Lo et al., 1995). The authors also showed that impedance decreased over time when Ca^2+^ is sequestered due to of disassembly of the tight junction (D’Angelo Siliciano and Goodenough, 1988). After EGTA is removed, tight junctions are assembled again and impedance is recovered (Figure 3G). Also, the authors could employ the chip to follow the behavior of airway epithelial cells cultured in an air-liquid interface (Figure 3H). The work established a standard and reproducible protocol for the fabrication of organ-on-chip systems with TEER-based sensing capabilities. This kind of platforms displays promising applications as TEER measurements are frequently used to follow epithelial integrity and differentiation in organs-on-chips (van der Helm et al., 2019; De Gregorio et al., 2020).

### Cell and Tissue Architecture

When cells constitute tissues, cell–ECM and cell–cell adhesions are orchestrated to achieve proper collective structure and organization. During tissue formation, cells selectively form bonds between them, change shape, migrate, and synthesize ECM (Gumbiner, 1996). In this context, materials with the ability to control topological and geometrical properties of cells are desired in order to induce the same cell architecture found in normal tissues. Due to the frequent complex 3D topological organization of cells in tissues, analyzing the response of single cells *in vivo* is challenging (Ladoux and Mège, 2017). Therefore, smart *in vitro* strategies are necessary to mimic as close as possible the conditions of cells in multicellular organizations. The incorporation of cell geometry aspects adds a layer of complexity to the design of biosensors, particularly if the nanometric topologies are also incorporated into the substrate material.

The ability to produce precisely engineered scaffolds can provide a way to control cell architecture during culture. Conventional cell cultures lack the ability to control cell spatial organization, therefore micropatterning techniques have been developed to create 2D patterns to control cell-substrate interactions and cell behavior. Micropatterning can be done by microcontact patterning (μCP), which consist in creating micro-stamps to deposit an “ink” material on it and print the material onto a substrate, resulting in a 2D pattern of the “ink” material on the surface (Alom Ruiz and Chen, 2007). The first micropatterning methods were developed in the late 60s (Carter, 1967; Harris, 1973). However, they became more available with the increased accessibility to photolithography techniques. The use of polydimethylsiloxane elastomer (PDMS) to prepare molds made the process easier, allowing to print patterns of proteins on substrates (López et al., 1993b), and to employ them to study cell adhesion in different patterns (Whitesides et al., 2001). There are other methods to create micropatterns on surfaces like photolithography (Bélisle et al., 2009; Kim et al., 2010), microwritting (López et al., 1993a), and microfiltration (Kailas et al., 2009), although μCP remains as one of the most common methods (Théry, 2010). Micropatterning was also successfully combined with nanopatterning to create substrate materials controlling topography aspects in the micro and nanometer range (Ren et al., 2017). New strategies were developed for orthogonally functionalized surfaces with two different adhesion ligands, controlling each nanodistribution independently (Polleux et al., 2011; Guasch et al., 2016; Yüz et al., 2018). Surfaces could be tailored with Au or TiO_2_ nanoparticles that act as anchor points of cell adhesive ligands. A similar approach was used to functionalize orthogonally micropatterns on a surface with two ligands with specificity for either α_5_β_1_ or α_V_β_3_ (Guasch et al., 2015). Using photolithography and metal sputtering, a micropatterned surface consisting of stripes of Au or TiO_2_ was synthesized. The different Au or TiO_2_ areas could be functionalized selectively by using integrin ligands with a thiol or phosphonic acid group respectively. These examples show how adhesive ligand distribution of a substrate can be precisely controlled to build micro and nanopatterned substrates.

The creation of micropatterns on different materials opens the possibility to introduce topographical features on surfaces with built-in sensing capabilities. Micropatterning can be applied to sensing materials in order to study the role of cell architecture on mechanobiology. Ribeiro et al. (2015) created Matrigel microtopographies on polyacrylamide substrates with embedded fluorescent beads to study the differentiation of cardiomyocytes from human pluripotent stem cells (hPSC-CM). The material design allowed to: (i) control the 2D cellular aspect ratio on the substrate; (ii) modulate the stiffness of the substrate; (iii) measure the contractile force of cardiomyocytes by traction force microscopy (TFM). hPCS-CM were cultivated on isolated rectangular micropatterns with different aspect ratios. The authors showed that myofibril alignment and contractile forces along the major axes of the cell were greatest in high aspect ratios (7:1) and physiological stiffness (10 kPa). These conditions indicated a more differentiated phenotype compared to cells growing on micropatterns with different aspect ratios and/or substrate stiffness. The results were also supported by the characterization of Ca^2+^ signaling, mitochondrial organization, and protein expression in the cells. The ability of this functional material to modulate cell aspect ratio, substrate stiffness, and force transduction constituted a powerful tool to control hPCS-CM differentiation.

However, despite the versatility of micropatterning techniques on 2D substrates, they fail to mimic *in vivo*-like 3D microenvironment and organization. Several strategies have been developed to create biomimetic 3D scaffolds like solid porous substrates (Lai et al., 2012), hydrogels matrixes (McKinnon et al., 2013), microconduits (Anderson et al., 2016), and microtracks (Kraning-Rush et al., 2013). The use of two photon polymerization allowed to create very complex 3D scaffolds for cell culture (Turunen et al., 2017). Nevertheless, the incorporation of controlled nanopatterning and built-in sensing capabilities is still a challenge in 3D culture matrixes. To create a 3D biosensor that could transduce cell behavior in 3D environments, Pitsalidis et al. (2018) designed an organic biotransistor, in which the conductive polymer poly(3,4-ethylenedioxythiophene) was doped with poly(styrene sulfonate) (PEDOT:PSS) to create a suitable scaffold for cell growth. The addition of collagen and single-walled carbon nanotubes (SWCNTs) for improving biocompatibility and electrical performance, respectively, was also studied. Then, the polymer scaffold was fixed between two electrodes in a chamber filled with aqueous solution containing a gate electrode. In these conditions, the organic polymer scaffold was in direct contact with two electrodes and indirectly with the gate through the aqueous medium. The system behaved as a transistor, the two electrodes induced a current that went through the scaffold, which depended on the gate voltage. Due to the porous nature of the scaffold, cells could attach and grow in it. Madin-Darby canine kidney cells II (MDCKII) proliferation inside the porous scaffold induced a decrease in transconductance, which allowed monitoring cell growth in real-time. Also, after 3 days of culture MDCKII cells exhibited less transconductance compared with telomerase immortalized fibroblast (TIF). The authors explained these results by the presence of a higher density of cell-cell junctions in MDCKII compared to TIF. Although this device is not yet fully biomimetic, it represents a step forward in creating electrochemical sensing platforms for 3D cell culture studies in real-time.

### Cell Adhesion Biomarkers

Biochemical signals have a critical role in tissue formation. They can be found free in the extracellular medium, embedded in the ECM or at the surface of other cells like the Notch signaling pathway (Chacón-Martínez et al., 2018). At the same time, cell differentiation is followed by the release of biochemical mediators like neurotransmitters in neurons (Kim et al., 2015; Kruss et al., 2017), hormones in endocrine cells (Lund et al., 2016; Hunckler and García, 2020), and ECM components (Mehlhorn et al., 2006; Zhang et al., 2020). Moreover, differentiation induces changes in cell metabolism that modifies the concentrations of intermediates in metabolic pathways (Quinn et al., 2013; Carey et al., 2015). The creation of active materials capable of detecting molecules released by cells, although is highly valuable, yet remains a challenge. This can be attributed to the fact that, in many cases, the sensor material is passivated by the same molecules released by cells (Spégel et al., 2007), and because of the complex mixtures of biochemicals that can interfere in the sensing process (Huang et al., 2011). Furthermore, high sensitivity should be provided, since often the concentration of the target molecule reaches low concentrations and for short periods of time (Amatore et al., 2008).

Electrochemical biosensors provide a versatile and sensitive means to probe the content of biological environments (Ding et al., 2008). Wang et al. (2018) created stretchable photocatalytically renewable electrodes for nitric oxide (NO) sensing by functionalizing PDMS films with a nanonetwork of Au nanotubes (NTs) and TiO_2_ nanowires (NWs). The Au NTs rendered electrochemical sensing performance, while the TiO_2_ NWs provided photocatalytic activity to recover the performance of the sensor after UV irradiation. In addition, electrochemical biosensors can measure neurotransmitters released by neurons, measuring the redox reaction of a neurotransmitter with the electrode. Kim et al. (2015) created a nanopatterned electrochemical sensor to monitor the dopaminergic differentiation of human neural stem cells (hNSCs). The nanopatterned surface showed increased cell adhesion and spreading of a dopaminergic cell line (PC12) and enhanced sensitivity toward dopamine compared to a planar gold or ITO electrode. Spatial resolution of neurotransmitter release could be obtained by using microelectrode arrays (MEAs). Wang et al. (2013) created subcellular MEAs ranging from 4 to 16 μm^2^ to record the release of dopamine across single cells and PC12 cell clusters.

**FIGURE 4:**
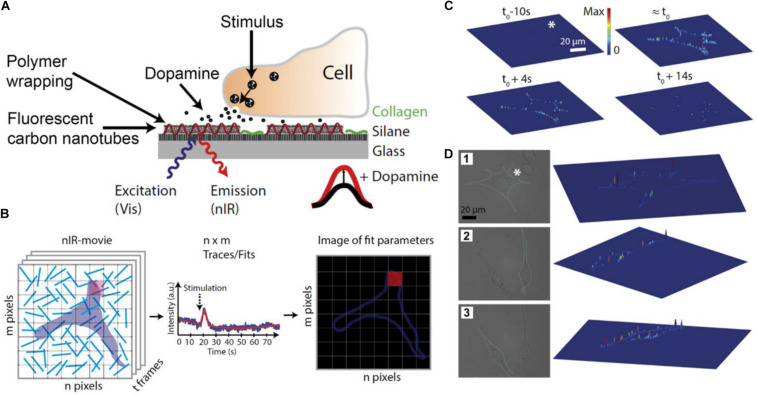
Detection of dopamine released by PC12 with nanosensor arrays. In **(A)**, a scheme of the biosensor surface where DNA-wrapped SWCNTs were immobilized on a glass surface. Surfaces were coated with collagen to facilitate cell adhesion. The DNA-wrapped SWCNTs modified their fluorescence spectrum as a consequence of non-covalent dopamine binding. In **(B)**, the scheme shows the analysis of experimental images. Each pixel of the images corresponded to a region containing one or more DNA-wrapped SWCNT nanosensors. Each pixel of the fluorescence movies produces a trace that contains information about the local dopamine concentration. A function was fitted to the data of each pixel to obtain the amplitude, width, and time of the signal. The fitted parameters can be represented in false-color images. In **(C)**, the image shows released dopamine profiles across the border of different cells. For this analysis, only pixels on the cell border were considered. On the top of **(C)**, 3D plots of fitted sensor signal responses at different times before or after stimulation (t_0_) are shown. Height and color of the 3D plot surfaces indicate the relative fluorescence change in pixels in the cell border normalized to the maximum fluorescent change in the same cell (values between 0 and 1). Results show that the maximum response is acquired at t_0_, then signal decreases. In **(D)**, images 1, 2, and 3 indicate different cells and their respective 3D plots. In this case, the height of 3D surface plots shows the maximum dopamine response obtained in each pixel, showing that dopamine is released at particular locations or “hot spots” on the cell membrane. Figures were adapted from Kruss et al. (2017) with permission. Copyright 2017 National Academy of Sciences.

In addition to sensors based on electrochemical transduction, sensors based on optical readout have been proposed to monitoring molecule release by cells (Kim et al., 2018; Dinarvand et al., 2019; Liu et al., 2020). Kruss et al. (2017) developed single-walled carbon nanotubes wrapped with short DNA sequences (DNA-wrapped SWCNT) to measure dopamine release from PC12 cells. These DNA-wrapped SWCNT displayed near-infrared fluorescence and changed their fluorescence emission spectrum in presence of specific organic molecules (Figure 4A). The authors optimized the DNA sequences to improve the selectivity and sensitivity toward dopamine. Then, sensors were immobilized on a glass surface and PC12 cells were seeded on it. PC12 cells dopamine release was measured by fluorescence microscopy. Surface immobilized DNA-wrapped SWCNT fluorescence emission depended on the local dopamine concentration on the surface. The authors recorded fluorescence images of the cells and divided the image into discrete pixels. A fitting algorithm was developed for the normalized fluorescence intensity traces of the pixel groups. Using this method the authors could localize transient peaks in fluorescence recordings due to dopamine exocytic events (Figure 4B). Results showed that PC12 cells secreted dopamine at determined exocytosis sites or “hot spots,” instead of a release of neurotransmitters at random locations on the membrane (Figures 4C,D). Notably, the authors demonstrated that the substrate functionalized with DNA-wrapped SWCNTs could render higher spatial resolution compared with MEAs and similar time resolution to that of cyclic voltammetry-based sensors.

Among existing materials for detecting the release of molecules from cells, metal nanopatterned substrates have a remarkable advantage. Metal nanostructured surfaces with plasmonic properties can enhance the Raman scattering of molecules, a phenomenon known as surface enhanced Raman scattering (SERS). Raman scattering occurs because of the inelastic scattering of photons by molecules. Due to their different vibrational modes, molecules produce a spectrum of Raman scattered light, which contains information about the chemical identity of the molecule (Kneipp et al., 2010). The Raman scattering is often weak, but when the molecules are very close to the metal nanostructured surface, their Raman scattering can be enhanced several orders of magnitude. The detailed mechanisms by which molecules enhance their Raman scattering near metal nanostructures are beyond the scope of this review, but the reader can refer to these excellent articles for more details (Haynes et al., 2005; Kneipp et al., 2010; Schlücker, 2014).

SERS is a promising strategy to analyze the chemical composition near a nanostructured surface in a non-invasive and sensitive way. Cells can be seeded on SERS substrates, and once they adhered and spread, their chemical composition near the surface can be studied by irradiating the substrate and measuring the Raman spectra. Over the years, a wide diversity of metal nanostructured surfaces have been created for SERS, such as nanoparticles attached to a surface (Freeman et al., 1995; Zhai et al., 2009; Lussier et al., 2016), nanopillars (Kang et al., 2015; Li et al., 2016), nanoholes (Abdelsalam et al., 2005; Luo et al., 2019), and others (Yüksel et al., 2017; Yao et al., 2020). A detailed review on the fabrication of SERS substrates can be found in Fan et al. (2011). El-Said et al. (2015) built a nanopatterned surface for *in vitro* monitoring of neural stem cell (NSC) differentiation. The authors electrochemically deposited Au on an ITO surface to obtain an array of Au nanostars. PC12 cells adhered and spread on this substrate, and could be electrically stimulated due to the conductive characteristics of the material. The Raman spectrum was recorded at different stages of the cells differentiation process and exhibited different peaks that could be attributed to the presence of specific functional groups in biomolecules. Although it is difficult to link the changes in Raman spectra with specific changes in cell biochemistry composition, the Raman spectra can be used as a fingerprint to follow the cell differentiation process in a non-invasive manner.

### Excitatory Cell Adhesion

Changes in membrane potential are a critical aspect of neurons and myocytes function. Action potentials travel through the membrane of these cells allowing communication of signals between different parts of the cell, eliciting the release of signaling molecules like neurotransmitters, and triggering contractile activity of muscle cells. Thus, proper cell electrical activity is an important characteristic of neural and cardiac differentiated tissues (Gunhanlar et al., 2017; Karbassi et al., 2020). Nowadays, neural and myocyte single-cell electrical activity can be measured by patch clamp techniques, optical imaging using genetically encoded or extrinsic fluorophores and substrate-integrated MEAs (Spira and Hai, 2013).

In particular, MEAs provide non-invasive monitoring of electrical activity and stimulation of multiple neurons *in vitro* and *in vivo* (Hutzler et al., 2006; Berdondini et al., 2009). At the beginning, electrodes were only capable of registering extracellular potential of cells (Thomas et al., 1972; Gross et al., 1977; Pine, 1980; Csicsvari et al., 2003), however, modifications of the material topology allowed to record the intracellular action potential. This could be achieved by the generation of protrusions of different shapes like mushrooms (Hai et al., 2010) and pillars (Robinson et al., 2012) where electrodes are inserted into the cell. Another configuration was created by Desbiolles et al. (2019). These authors fabricated a surface containing nanovolcanoes with an electrode in their interior. Cells fused spontaneously with the nanovolcano allowing the electrodes to be in contact with the intracellular medium (Desbiolles et al., 2019). Other approaches include kinked nanowires (Tian et al., 2010) or nanotubes (Duan et al., 2012). In these cases, the nanostructures allow a field-effect transistor to gain access to the intracellular medium. Protrusive nanostructures disrupt the membrane and insert into the cytoplasm spontaneously (Desbiolles et al., 2019), by electroporation (Xie et al., 2012), or by chemical functionalization (Duan et al., 2012).

Planar patch clamp chips are other type of substrates used to measure intracellular potential, constituting a protrusion-free approach. Using this strategy, Martina et al. developed a planar substrate with holes from 2 to 4 μm in diameter that connected to a microfluidic channel under the surface (Martina et al., 2011). Neurons adhered to the substrate and spread, covering the holes. Then, negative pressure was applied in the channel, which breaks the cell membrane and connects the cytoplasm with the microfluidic channel, similarly to a whole-cell patch-clamp configuration. Thus, the intracellular potential could be registered through the microfluidic conduits.

**FIGURE 5:**
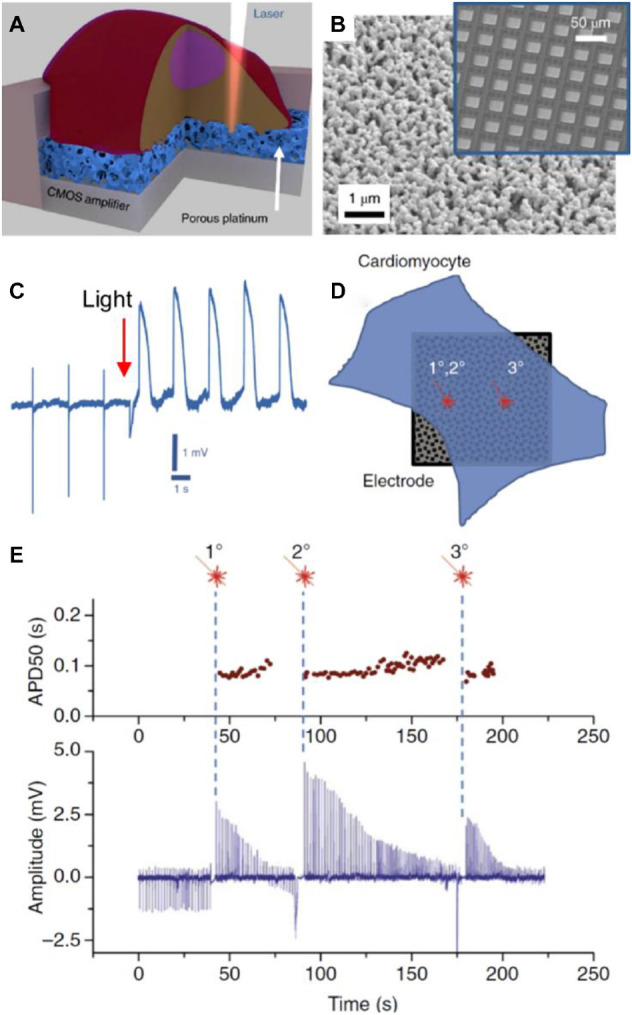
In **(A)**, a scheme of the porous platinum electrode on the CMOS amplifier. Due to the structure of the platinum electrode, cell poration can be done with light (opto-poration). In **(B)**, the image shows a surface electron microscopy image of the porous platinum surface of an electrode. In the inset, an image of the CMOS-MEA employed by the authors is depicted. In **(C)**, a recording of the electrode signal where the instantaneous transition from the extracellular to intracellular recording takes place. In **(D)**, the image represents the opto-poration of a cardiomyocyte in the same spot twice (1° and 2°) or in a different spot (3°). Results of electrical activity are shown in **(E)**. At the top, action potential duration at 50% amplitude (APD50) as a function of time after opto-poration. At the bottom, intracellular signals as a function of time. Signal amplitude decreased as a function of time due to membrane re-sealing. However, action potential duration (APD50) remained at the same value, no matter if opto-poration was performed twice on the same spot or other places of the cell. Figures were adapted from Dipalo et al. (2018) with permission Copyright 2018 Springer Nature.

Usually, electrodes in MEAs are made of metallic conductors like gold, titanium nitride, platinum, and alloys like iridium oxide (IrOx). Electrode surface can be modified with porous materials from platinum, gold nanostructures, CNTs, and conductive polymers like poly(3,4-ethylenedioxythiophene) (PEDOT) (Obien et al., 2015) to increase the effective surface area of electrodes. In the last years, the use of complementary metal-oxide-semiconductor (CMOS) technology provided an increase of electrode density in MEAs (Obien et al., 2015). Despite the promising sensitive capabilities of protruding conductive electrodes to measure intracellular action potential, their effects on cells are not fully characterized. For example, it has been reported that protrusions that insert into cells could alter intracellular trafficking (Zhao et al., 2017). In the case of non-protrusive strategies like planar patch clamp chips, the measurement time could be limited by the perfusion of microfluidic liquid into the cell. Because of this, a different approach was employed by Dipalo et al. (2018) consisting of MEAs made of platinum or gold porous electrodes (Figures 5A,B). The electrodes acted as plasmonic antennas, which under infrared light illumination generated acoustic waves that transiently porate the cell membrane at the illuminated spot, a process named “optoacoustic poration.” A platinum porous electrode was placed on top of the aluminum surface of the CMOS-MEA electrodes. Cardiomyocytes derived from human-induced pluripotent stem cells (hiPSCs-derived cardiomyocytes) were cultured on the MEA. After optoacoustic poration of cardiomyocytes the intracellular action potentials could be recorded (Figure 5C). Interestingly, membrane potential recordings were not affected by repeated optoacoustic porations on the same spot or different spots on the same cell (Figures 5D,E). The material took advantage of the nanostructured properties of the porous electrode to facilitate the access to the intracellular medium and provided a robust and reliable way to measure intracellular action potentials.

### Measurements of Cell-Generated Forces

Upon adhesion, cells generate contractile forces to their supporting material. On materials of soft composition, the forces transmitted from the cells cause substrate deformation. This phenomenon can be exploited to assess the link between the mechanical properties of cells and the stiffness of the extracellular environment. In the beginning, forces were estimated by counting wrinkles as a consequence of material deformation by the cell (Harris et al., 1980). Forces could be quantified by a calibration curve created by correlation of forces of known magnitude with the length of the wrinkles they produce (Burton and Taylor, 1997). However, this methodology was limited in terms of spatial and temporal resolution as wrinkles are usually larger than cells, they develop slowly, and are intrinsically non-linear (Dembo and Wang, 1999). A major improvement was introduced in the work of Dembo and Wang (1999), where fluorescent beads were embedded in the soft polymer layer on which cells grow, establishing the basis of traction force microscopy (TFM). In classic TFM, forces exerted by the cells on the soft material are calculated by tracking the displacement of the tracer particles by fluorescent microscopy. An image of the substrate in a stress-free reference state is compared with the image of the substrate in the presence of cells.

Over the last few years, TFM has been implemented on diverse elastic materials like silicone, polyacrylamide, and polyethylene, being polyacrylamide one of the most used due to its transparency and elastic properties (Mohammed et al., 2019). Regarding elasticity, substrate materials in TFM are generally linearly elastic, i.e., stress is directly proportional to strain, which facilitates force calculation. However, the ECM is a fibrous network, and therefore elasticity is not linear. For a more detailed description of the mechanical properties of the ECM see the recent review from Polacheck and Chen (2016). The first attempts to measure forces in non-linear elastic biopolymers were implemented by Steinwachs et al. (2016). Often, substrate materials are coated with ECM components like collagen, fibronectin, laminin, or peptide mimetic ligands of these proteins to provide cell-adhesion sites on the substrate. A detailed protocol and guideline to perform TFM can be found in a manuscript from Plotnikov et al. (2014).

**FIGURE 6:**
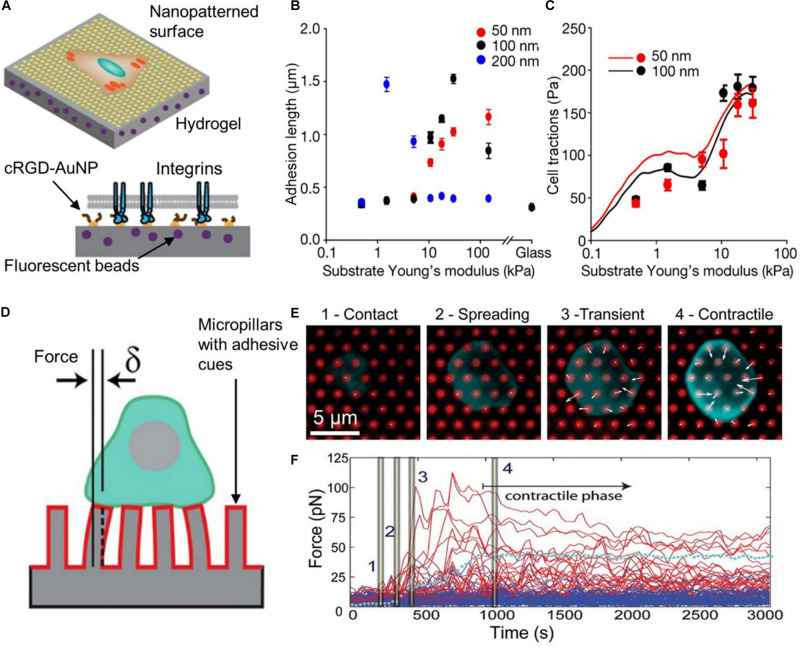
Substrate materials for measuring cell-exerted forces. In **(A)**, the scheme represents the nanopatterned substrate employed by Oria et al. (2017). Hydrogels with different stiffness and coated with AuNP at different distances were employed. AuNP were functionalized a cRGD-based ligand (integrin ligand). Due to steric hindrance only one integrin could bind to each AuNP. The length of FA (adhesion length) was measured using cells that expressed GFP-paxillin. In **(B)**, results show that greater FA were assembled on soft substrates with high spacing between ligands (200 nm, blue dots). On stiffer substrates, longer FA were detected at shorter ligand spacing (50 nm, red dots). In **(C)**, the graph shows averaged cell tractions as a function of substrate stiffness. The continuous lines represent the predictions of the model created by the authors to explain the results. Figures in **(A–C)** were adapted from Oria et al. (2017) with permission. Copyright 2017 Springer Nature. In **(D)**, a scheme of a T cell on an array of elastomer pillars is presented. These pillars were coated with antibodies that activated the TCR and the CD28 (red coating on the pillars). Forces exerted by cells were calculated by measuring the pillar displacement (δ). In this type of material, pillar displacement is proportional to the force applied to them (for small displacements). In **(E)**, the images show different phases of the T cell interaction with the substrate. White arrows indicate the direction of forces and their magnitude. An arrow of the same length of the 5 μm scale bar represents a force of 250 pN. In **(F)**, the graph shows the force applied to individual pillars as a function of time. Red lines represent the forces exerted on pillars in contact with the cells whereas blue lines indicate forces on pillars not in contact with the cell. The dotted cyan line represents the average pillar force within the same cell. Time points indicated with bars in the trajectory correspond to the images in **(E)**. Figures in **(D–F)** were adapted from Bashoura et al. (2014) with permission. Copyright 2014 National Academy of Sciences.

In a recent work, Oria et al. (2017) fabricated polyacrylamide hydrogels of different stiffness embedded with fluorescent nanobeads and decorated with a quasi-hexagonal array of gold nanoparticles on their surface (Figure 6A). Nanometer scale distribution of integrin ligands (cyclic arginine-glycine-aspartate, cRGD) was achieved by functionalization of the gold nanoarray, while the tracking of the fluorescent beads allowed the measurement of cell forces. The created substrates offered a versatile platform for studying how cells sense spatial and physical information at the nanoscale. Interestingly, the authors showed that ligand spacing and substrate stiffness had opposite effects on cell behavior. Increased adhesion was produced at long ligand spacing on less rigid surfaces (200 nm, 1.5 kPa) or at short ligand spacing on more rigid surfaces (50 nm, 30 kPa). The authors measured the length of FAs on cells that expressed a paxillin labeled with a green fluorescent protein (GFP-paxillin), which was an indicator of the maturation of the FA. Results showed how adhesion depended on the ligand spacing and surface stiffness (Figure 6B). At higher substrate stiffness, the cell exerted higher tensile forces on the surface (Figure 6C). Moreover, cell adhesion was dependent on whether ligand distribution was ordered or not (homogenous ligand spacing or random distribution). These results ruled out a molecular-rule hypothesis in which there is an optimal ligand spacing for FA assembly and growth. On the contrary, the authors explained their results by a model that takes into account pulling forces exerted by myosin on actin filaments, a force threshold that triggers FA growing, and a maximum integrin recruitment in the FA. On soft surfaces, ligand spacing has to be long, so that actin-pulling forces are distributed on fewer integrins. Thus, the force on each integrin is high enough to trigger FA growing. As more rigid surfaces were employed, FA can grow until the maximum number of integrins in the cluster is reached. If stiffness is too high, the pulling force exerted by the cell overpass the FA binding force to the substrate and adhesion collapse. This work is an exquisite example of how materials can be engineered to mimic different properties of the cellular environment, and at the same time provide sensitive means for probing cell mechanics.

Recently, TFM substrates were improved by the generation of precise arrays of fluorescent quantum dots inside a silicone elastomer by electrohydrodynamic nanodrip-printing. The creation of controlled arrays of fluorescent particles removes the necessity of an image of the field after cell detachment (Bergert et al., 2016). Moreover, the use of super resolution fluorescence microscopy increased the resolution of the force map on the substrate (Colin-York et al., 2016; Stubb et al., 2020). However, calculation of the force maps from the microscopy images is not a trivial process and requires complex algorithms. For a more detailed review on this matter see Style et al. (2014), Schermelleh et al. (2019).

TFM-optimized elastic materials have been created to study mechanobiology in 3D. The deconvolution in 3D of forces exerted by the cell on a surface (Maskarinec et al., 2009; Legant et al., 2013) or inside 3D matrixes (Legant et al., 2010) was developed. Although the deconvolution of the force map in 3D environments is still challenging (Polacheck and Chen, 2016), these approaches hold great promise for measuring cell forces and mimic the ECM in geometries that more closely resemble those found in normal tissues. Vorselen et al. (2020) created a sophisticated version of 3D TFM by using uniform hydrogel particles with tunable size and stiffness. These particles were incubated in the cell medium and suffer deformation as a consequence of the forces applied to them. A computational method was developed by the authors to infer the mechanical forces from the deformation of the hydrogel particles. Thus, the pressure exerted by the cell to the particle could be measured with high resolution. This approach moves away from the classical TFM concept as hydrogel particles are not fixed around the cells but free to move and interact with them. For example, the authors could measure the spatial distribution of forces applied by a macrophage that phagocyted a hydrogel particle. Besides, particles could be tailored with pMHC ligands for the T cell receptor (TCR) of lymphocytes and I-CAM. These ligands induced the adhesion of cytotoxic T lymphocytes to the particle, simulating the interaction of T lymphocytes with their target cells, thus allowing to monitor forces induced by the T cell. Remarkably, the authors introduced hydrogel particles as micrometer tension probes breaking the general concept of TFM limited to 2D surfaces.

Other class of elastic materials have been developed to study cell mechanical properties and behavior. The use of micropillars to measure cell pulling forces was first introduced by Tan and coworkers (Tan et al., 2003). In this strategy, an array of cylindrical micrometer-scale cantilevers called micropillars is fabricated on polyacrylamide or polydimethylsiloxane (PDMS) substrates. The micropillars top is functionalized with ECM molecules, allowing cells to adhere on them. Therefore, when cells spread and maturate, they exert forces on the micropillars supporting them. The magnitude of these forces can be calculated from the micropillar displacement from their undeflected positions. This has the advantage that there is no need for cell detachment processes as in classical TFM (Roca-Cusachs et al., 2017; Banda et al., 2019). Besides, force calculation is simpler than with TFM, because small deformations follow a linear regime: micropillar deflection is directly proportional to the force (Du Roure et al., 2005). A third advantage is that micropillar stiffness can be modulated to some extent by modifying pillar geometry (Saez et al., 2007). This feature was exploited to create a wide range of stepped gradients by combining different pillar geometries (Lee et al., 2015). However, a major limitation of pillar arrays is that the substrate is not continuous. Cells can bind only to discrete spots (the top of the pillars) which can affect the morphology of cell-ECM adhesion.

Researchers have been exploring the use of micropillars with magnetic properties to exert controlled forces on cells (Sniadecki et al., 2007; Le Digabel et al., 2011) or the generation of nanopatterns on top of the micropillar surface to control ligand spacing and the selective binding of integrin types (Rahmouni et al., 2013). Recently, Bashoura et al. (2014) assessed the role of receptors involved in the interactions of T cell lymphocytes with APC. A micropillar pattern was coated with antibodies that bind to the TCR or the CD28 proteins to mimic the activation exerted by the ligands of these proteins (Figure 6D). Thus, the micropillar surface simulates the membrane activation by the APC. Pillars were also coated with fluorescent molecules to facilitate the force map generation. Using antibodies or ligands for the TCR or CD28, the authors could demonstrate that T lymphocytes exert pulling forces through the TCR (Figures 6E,F), whereas CD28 on its own does not mediate forces. Instead, CD28 is important for signal transduction.

Other approaches have been developed where forces can be measured without the need for soft deformable substrates. In 2012, Salaita’s group developed a sensitive approach to spatially and temporally map forces exerted by cells. Similar to protein tension probes expressed by cells (Grashoff et al., 2010), they implemented molecular probes as force transducer sensors (Stabley et al., 2012). The sensor consisted of a flexible polyethylene glycol (PEG) linker covalently bound to a ligand at one terminus and anchored onto a surface. The ligand and the surface were functionalized with a fluorophore and a quencher molecule, respectively. In a resting state, the linker is in a collapsed conformation, allowing the fluorophore to be quenched due to proximity with the quencher. When a force is applied to the probe, the linker stretches, separating the fluorophore from the quencher and increasing the fluorescence yield of the probe. Thus, knowing the quenching efficiency at a particular spot on the surface, it is possible to determine the distribution of collapsed/extended probes. As a proof of concept, the authors used an epidermal growth factor (EGF) ligand to map forces associated with initial uptake and trafficking of the EGF receptor (EGFR) upon binding to its cognate ligand. This work was motivated by the need for functional materials that can surpass the limitations of TFM in terms of sensitivity, spatial, and temporal resolution. Later on, the same group applied a similar concept to quantify the innate forces involved in the binding of TCR to the pMHC (Liu et al., 2013, 2016). The force sensor consisted of a DNA hairpin labeled with a fluorophore-quencher pair immobilized onto a gold nanoparticle (AuNP). Both the molecular quencher and AuNP supported on glass quench the fluorophore by FRET and plasmon effect, respectively. The dual quenching mechanism provided increased sensitivity and lower background signal.

**FIGURE 7:**
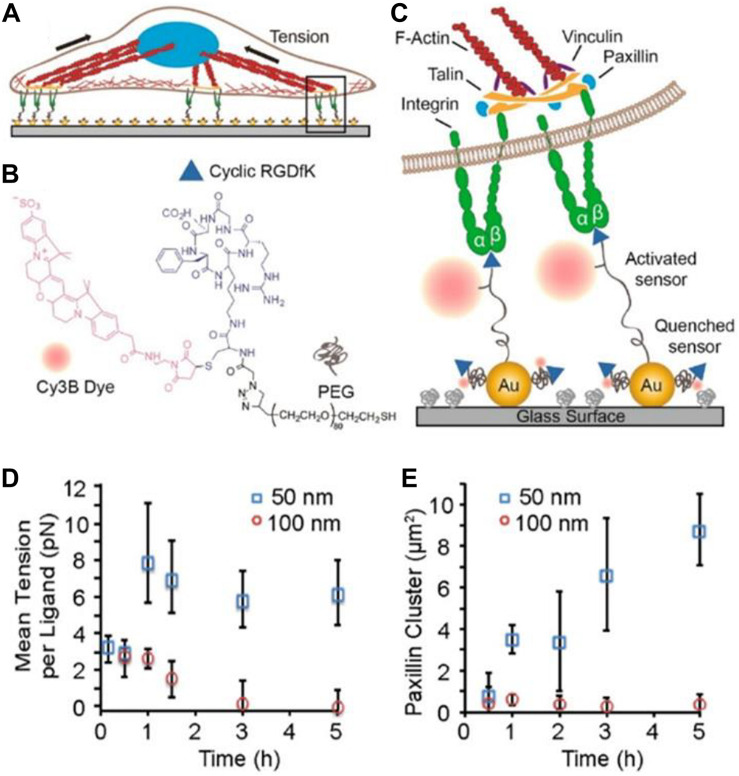
Molecular fluorescence tension microscopy substrates. In **(A)**, the image shows a drawing of a cell adhered on the nanostructured biosensor surface. In **(B)**, the chemical structure of the tension probe synthesized by the authors is shown. The probe is composed of three main components, a cyclic RGDfK which constitutes the integrin ligand (represented by a blue triangle), a Cy3B dye (represented by a red dot), and a PEG chain with a thiol group at the end (represented by a gray line). The probe binds to the AuNP through the thiol group and acquires a collapsed conformation in the absence of pulling forces on the probe. Each AuNP has on average 2.5 probes. The image in **(C)** shows the scheme of the molecular probe. In a rest position the probe acquires a collapsed conformation where the Cy3B fluorescence is quenched by the AuNP. Upon exertion of pulling forces by the cell, the linker stretches and the fluorescent probe is no longer quenched. In **(D)**, the graph shows the mean tension per ligand across one entire cell on substrates coated with AuNP at different interparticle distances. After 1 h only cells on surfaces with high ligand density (50 nm spacing) could exert forces higher than 5 pN. The graph in **(E)** shows the GFP-paxillin cluster size (which indicates FA size) as a function of time. The increase in FA size was correlated with high tension as observed in **(D)**. Figures were adapted from Liu Y. et al. (2014) with permission. Copyright 2014 American Chemical Society.

The use of molecular tension probes was also implemented on nanopatterned surfaces to study the molecular biophysics of integrin ligand clustering (Liu Y. et al., 2014) (Figure 7A). Nanopatterned surfaces were prepared to create an array of AuNP, in which the distance between AuNP is precisely controlled (between 30 and 300 nm). Molecular tension probes were bound to AuNP on one extreme and had an integrin ligand [cyclic Arg-Gly-Asp-dPhe-Lys, c-(RGDfK)] on the other. A Cy3B fluorophore was located next to the integrin ligand (Figure 7B), so in the resting state, the fluorescence was quenched by the plasmon of the AuNP via nanometal surface energy transfer (NSET) (Figure 7C). The authors studied the adhesion of NIH/3T3 fibroblasts that expressed a recombinant GFP-paxillin (a protein expressed at the FA). A relationship between integrin ligand density and the magnitude of forces exerted by NIH/3T3 fibroblast was found. At low ligand density (100 nm of ligand spacing) cells showed less adhesion, smaller FA after 30 minutes, and lower pulling forces compared to cells cultured at higher ligand density (50 nm of ligand spacing) (Figure 7D). In the latter case, forces exerted by cells increased until a maximum value (at 1 h) after which forces remained constant. This could indicate that integrin ligand density has to be high enough to harness actin and myosin driven tension, which is necessary for FA maturation. Using cells expressing GFP-paxillin, the authors could measure the size of FA (Figure 7E). Results showed a similar behavior of the forces and FA size as a function of time. At higher ligand densities, cells exerted higher forces and assembled bigger FAs.

## Summary and Future Perspectives

In the last years, advances in material synthesis have allowed the incorporation of sensing capabilities into cell substrate materials, which expanded the experimental information obtained from cell adhesion studies. This trend continued as novel synthetic strategies to tailor specific properties to materials were in constant development, leading to improvements in terms of sensitivity, spatial, and time resolution. In addition, modulation of topographical and chemical features allowed more control over cell functions. Surface nanopatterning was crucial to reproduce the physicochemical characteristics of the cellular microenvironment, creating accurate synthetic ECM analogs. Novel synthetic strategies together with sophisticated analysis techniques contributed to address important aspects of the mechanism by which cells interact with their microenvironment. For instance, accurate localization of fluorescent nanotracer inside soft elastic materials was crucial to spatially and dynamically map cell forces by stimulated emission depletion (STED) microscopy (Bergert et al., 2016; Stubb et al., 2020). Adding multiple features to the functional material provides more realistic physiological conditions and higher information output as demonstrated using precisely distributed nanometer-scale arrays of ECM ligands on TFM substrates of variable rigidity (Oria et al., 2017). 3D soft architectures with programmed physicochemical properties incorporating a transducer element were also demonstrated (Pitsalidis et al., 2018; Vorselen et al., 2020), which constitute a necessary step towards a new paradigm for *in vitro* studies of cell processes.

3D synthetic microenvironments provide a platform for cell culture and cell analysis *ex vivo* where cells behave more natively (Weigelt et al., 2014). Advances in 3D culture platforms that merge biosensing capabilities with sophisticated biochemical and biophysical properties as those found in the native ECM allow the real-time study of mammalian tissues (Shamir and Ewald, 2014). This type of stimuli-responsive functional materials would be ideal toward understanding how cells change their phenotype and acquire specific functions as a consequence of the cues from the environment. This is an important aspect of tissue physiology, and a critical step to understanding alterations that lead to tissue pathophysiology (Bhatia et al., 2014; Pickup et al., 2014; Gaetani et al., 2020). In this context, biomimetic 3D biosensors for cell culture will increasingly contribute to physiology, histology, and physiopathology studies.

3D matrices that combine essential biophysical and biochemical aspects of the native cellular microenvironment with biosensing features will also bring benefits to the field of tissue regeneration and healing. A major goal in this field is to improve the biointegration of orthopedic and dental implants with the surrounding tissues. During decades, numerous surface modification strategies based on chemical coatings and nanotopographical features were developed (Mas-Moruno et al., 2019). However, despite the research efforts, implants still nowadays fail at an unacceptable rate (Raphel et al., 2016). This, in part, is due to the lack of critical information about the mechanisms governing the material biointegration process. In this perspective, ECM mimetics able to monitor cell adhesion dynamics quantitatively in real time hold great promise toward a rational design of ‘smart’ implants that possess cell-instructive characteristics.

A further challenge should be devoted to the development of novel approaches that could provide information in real time of the cell behavior *in vivo*. Mammalian tissues and organs are particularly difficult to study by direct optical observation (Shamir and Ewald, 2014). This is the case for a number of diverse 3D culture formats including organoids (Simian and Bissell, 2017). Electrochemistry could provide the means to accomplish this goal. Conducting polymers, for instance, are excellent building blocks for the creation of electrically responsive hydrogels (Zhang et al., 2019; Yang et al., 2020), although improvements in conductivity are desired to achieve highly sensitive cell sensing. Alternatively, the integration of hydrogels with conductive nanomaterials could overcome those limitations (Li et al., 2018). A fundamental requirement is to maintain not only the morphology of the hydrogel, but also the electrostatic and biocompatible properties, which will be essential for observing cells on a controllable nature-loyal microenvironment.

The electronic industry had advanced in the creation of complex circuits in very small dimensions. Adapting these advances to the field of cell biology emerges as a promising way to improve biosensor sensing capabilities. For example, the incorporation of CMOS-based MEAs in biosensors increased the density of electrodes on a surface (Obien et al., 2015), and with it the spatial resolution. Moreover, electrochemical biosensors can enhance biocompatibility by the creation of flexible electrodes (Song et al., 2020).

Finally, substrates with built-in sensing capabilities are suited for multiparametric cell monitoring. For example, transparent electrodes allow simultaneous microscopy observation and ECIS recordings (Pallarola et al., 2017a; Parviz et al., 2017), whereas nanostructured conductive substrates allow the implementation of ECIS and SERS (Zong et al., 2015). Often the result is more than the sum of its parts; SPR conductive substrates allowed the creation of electric impedance microscopy which added spatial resolution to ECIS measurements (Wang et al., 2011). Multiparametric approaches increase the information output from cell culture experiments. A further challenge will be the creation of computational models and simulations that could help to interpret and understand the multi-scale information obtained by multiparametric biosensors.

## Author Contributions

DP and NS designed the content of the manuscript. All the authors performed the literature survey, wrote the manuscript, and edited and reviewed the manuscript before submission. NS and DP prepared the figures.

## Conflict of Interest

The authors declare that the research was conducted in the absence of any commercial or financial relationships that could be construed as a potential conflict of interest.
